# Supplementary material for: The Systems Biology Research Tool: evolvable open-source software
Source: BMC Syst Biol. 2008 Jun 29;2:55. doi: 10.1186/1752-0509-2-55 (PMC2446383; doi:10.1186/1752-0509-2-55)
Supplement: Additional file 1 — SBRT Archive. An archive of the current version of the Systems Biology Research Tool. [file 1752-0509-2-55-S1.zip › sbrt-1.4.0/doc/developers_guide/api/sbrt/ext/R/stat/RSpearman.html]

RSpearman


|  |  |  |
| --- | --- | --- |
| **Overview** \| **Package** \| **Class** \| **Use** \| **Tree** \| **Deprecated** \| **Index** \| **Help** | |  |
| **PREV CLASS**   NEXT CLASS | **FRAMES**    **NO FRAMES**     **All Classes** |  |
| SUMMARY: NESTED \| FIELD \| CONSTR \| METHOD | DETAIL: FIELD \| CONSTR \| METHOD |  |


---


## sbrt.ext.R.stat Class RSpearman<V>

```
java.lang.Object
  sbrt.ext.R.stat.RCorrelator<V>
      sbrt.ext.R.stat.RSpearman<V>
```

**Type Parameters:**: `V` - the variable type.

**All Implemented Interfaces:**: Correlator<V,RCorrelator.Result>

---

``` public final class RSpearman<V> extends RCorrelator<V> ```

This class computes Spearman rho values using R.

**Author:**
:   This class was written and documented by
    Jeremiah Wright while in the Wagner lab.

---

**Nested Class Summary**

**Nested classes/interfaces inherited from class sbrt.ext.R.stat.RCorrelator**

`RCorrelator.Result`


**Constructor Summary**

`RSpearman()`


| **Method Summary** | |
| --- | --- |
| `protected  java.lang.String` | `getCorrName()`             Returns `spearman`. |

**Methods inherited from class sbrt.ext.R.stat.RCorrelator**

`corr, corr, corr, main`

**Methods inherited from class java.lang.Object**

`clone, equals, finalize, getClass, hashCode, notify, notifyAll, toString, wait, wait, wait`

**Constructor Detail**

### RSpearman

```
public RSpearman()
```


**Method Detail**

### getCorrName

```
protected java.lang.String getCorrName()
```

:   Returns `spearman`.

    :   **Specified by:**: `getCorrName` in class `RCorrelator<V>`
    :   **Returns:**: `spearman`.


---


|  |  |  |
| --- | --- | --- |
| **Overview** \| **Package** \| **Class** \| **Use** \| **Tree** \| **Deprecated** \| **Index** \| **Help** | |  |
| **PREV CLASS**   NEXT CLASS | **FRAMES**    **NO FRAMES**     **All Classes** |  |
| SUMMARY: NESTED \| FIELD \| CONSTR \| METHOD | DETAIL: FIELD \| CONSTR \| METHOD |  |


---
